# Experimental Study on the Application of Cementless Material with Industrial By-Products to Steam-Cured Precast Concrete Products

**DOI:** 10.3390/ma15217624

**Published:** 2022-10-30

**Authors:** Minoru Hata, Makoto Sato, Shingo Miyazawa

**Affiliations:** 1 Nippon Hume Corporation, Kumagaya 360-0161, Japan; 2 Tokyo Metropolitan Sewerage Service Corporation, Tokyo 100-0004, Japan; 3 Department of Innovative Engineering, Faculty of Engineering, Ashikaga University, Ashikaga 326-8558, Japan

**Keywords:** fly ash, ground granulated blast furnace slag, precast concrete, steam curing, compressive strength, durability, load-bearing capacity

## Abstract

The purpose of this study was to apply a cementless binder using industrial by-products (fly ash, ground-granulated blast furnace slag, and silica fume) to precast concrete products. In this binder, calcium hydroxide was included as an alkali stimulant but Portland cement was not included. Experiments on the compressive strength and durability of this type of material were conducted and its applicability to precast concrete products was investigated using full-scale specimens. The experiments proved that high-temperature steam curing is effective at obtaining strength development and that compressive strength can be expressed as a linear function of the binder–water ratio. Experimental results of chloride ion diffusion coefficient and sulfuric acid resistance suggested that the proposed material has higher resistances than conventional cement concrete against these deterioration factors. It was also demonstrated that full-scale specimens of a box culvert and a centrifugally compacted pipe using this type of material have almost the same load-bearing capacity and deformation performance as those using conventional cement concrete. It is believed that the proposed material could be used as a construction material instead of cement concrete, contribute to reducing CO_2_ emission, and increase the reuse of industrial by-products.

## 1. Introduction

The population of workers in Japan’s construction industry is decreasing, which correlates with the country’s declining workforce due to the decreasing birthrate and aging society. Moreover, a large part of the infrastructure built during the period of rapid economic growth has deteriorated, leading to calls for countermeasures. In these circumstances, demand has increased for precast concrete products that provide high quality, high durability, and high construction efficiency. Meanwhile, reducing the burden on the global environment has become a pressing issue in the field of concrete technology. Effective solutions to this problem include reductions in the amount of cement used, which cause large CO_2_ emissions during production, and the use of industrial by-products as concrete materials.

Therefore, attempts have been made to use large amounts of industrial by-products as supplementary cementitious materials in order to suppress the cement content of concrete. These include a method with significantly high ratios of ground granulated blast furnace slag (BFS) and fly ash (FA) in place of cement. In recent years, there have been studies in which the ratios of these materials in the binder were drastically raised to eliminate cement for precast concrete products. Under these circumstances, many researchers have studied the alkali-activated material known as geopolymer [1,2], which is a solidified product produced by condensation polymerization reaction of alkali–silica solution, such as water glass and alumina-silica powder, e.g., FA. There have been many research reports on this kind of material, and some have demonstrated sufficient mechanical properties for structural materials [3,4]. It is also believed that they have higher durability against salt penetration and chemical attacks than conventional cement concrete [5,6,7]. Fundamental research on the durability of alkali-activated materials in a sewer environment was also reported [8].

Regarding applications to concrete structures, they have been applied to the street–sideway boundary blocks [9] and sleepers [10]. The largest application may be for airport runway materials in Australia [11]. Alkali-activated materials produced by steam curing and autoclaved curing, which are generally applied to conventional precast concrete products, were also studied; it was reported that rapid strength development is possible by these high-temperature curing methods [12,13].

However, the production of the above-mentioned geopolymers generally requires the handling of concentrated aqueous alkali solutions, which are corrosive, viscous, and difficult to handle. Furthermore, the reaction of an alkali metal source with silicate powder is essentially needed in addition to the mixing process with water. Therefore, it is desirable that this kind of material be user-friendly, such as conventional cement concrete, and could be manufactured by simply adding water to the powder [14]. As a research example of such material development, the effect of calcium hydroxide (CH) as a stimulant on properties of fresh and hardened properties of cementless mixtures with FA, BFS, and anhydrous gypsum was reported [15]. It was also reported that CH and an expansive additive were used as alkali stimulants to harden a mixture with BFS in order to produce segments for shield tunneling [16]. Currently, however, there are very few application examples of these types of alkali-activated materials to products and structures. In particular, the applications of these kinds of materials to steam-cured concrete products have rarely been reported.

Under these backgrounds, the goal of this study involves the application of alkali-activated cementless material to steam-cured precast concrete products. Among various types of alkali-activated materials, this research focuses on user-friendly mixtures containing FA and BFS that can be used simply by adding water to the powder and mixing [14]. In this paper, the proposed alkali-active binder is referred to as industrial by-product material (IBPM). In view of the application to various kinds of precast products with different compressive strengths of concrete, the relationship between water–binder ratio and compressive strength of steam-cured IBPM concrete was experimentally investigated within a strength range of 40 to 80 N/mm^2^. Its durability was also experimentally examined in terms of the chloride ion-shielding capability and sulfuric acid resistance required for precast concrete products depending on their uses and environmental conditions. Furthermore, mock-up specimens of box culverts and Hume pipes using IBPM concrete were prepared to evaluate the load-bearing performance.

## 2. Materials, Mix Proportions, Mixing, and Curing

### 2.1. Materials and Composition of IBPM

**Figure 1:**
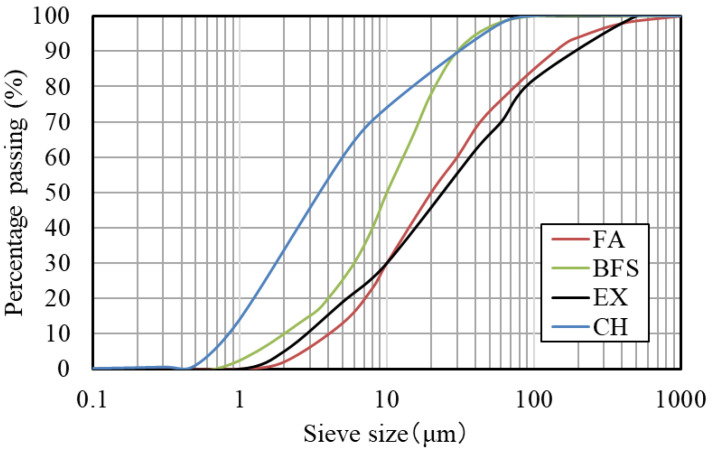
Particle size distribution of materials for IBPM.

**Figure 2:**
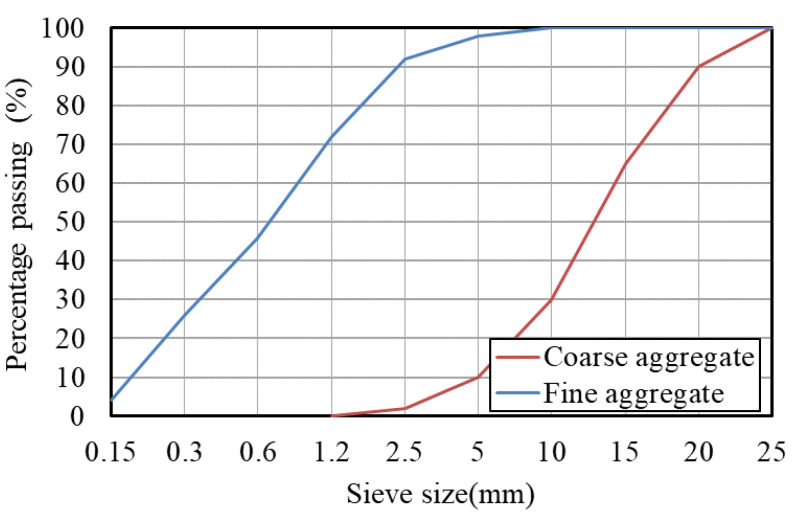
Particle size distribution of aggregates.

**Figure 3:**
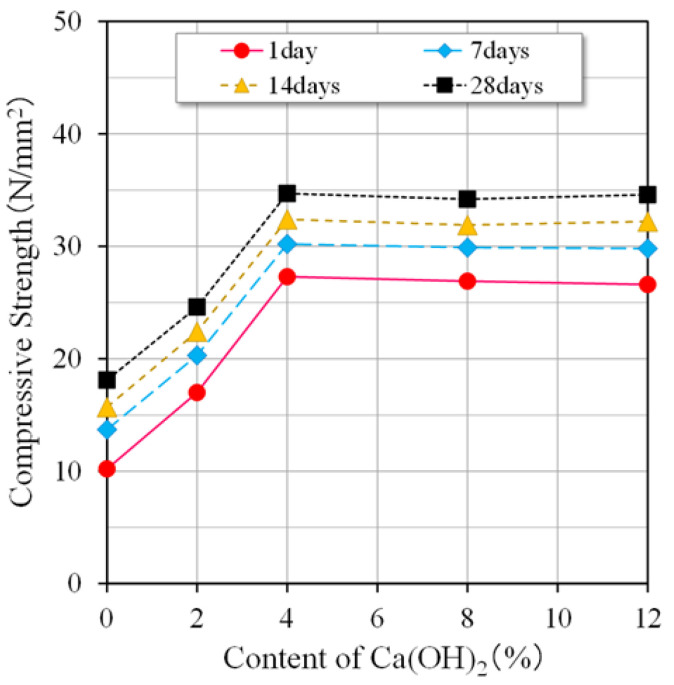
Effect of Ca(OH)_2_ content on the compressive strength of IBPM mortar.

As listed in Table 1, FA, BFS, and silica fume (SF) were used as the industrial by-products for IBPM concrete. Calcium hydroxide (CH) was used as a stimulant. An expansive additive (EX), which is generally used for reducing shrinkage cracking of hardened cement concrete, was also used. Table 2 shows the chemical compositions of the materials for IBPM, where the total contents do not add up to 100% due to unanalyzed minor components. Figure 1 shows the particle size distributions of FA, BFS, EX, and CH. The average diameter of the SF particle calculated from a specific surface area was 0.14 μm. Crushed stone and crushed sand (as given in Table 3 and Figure 2) were used as coarse and fine aggregates, respectively. The maximum size of the coarse aggregate was 20 mm. Polycarboxylate-type high-range water-reducing admixture (Sp) was used as the chemical admixture. For convenience, when using IBPM concrete in precast concrete factories, the authors intended to handle the above-mentioned five materials as premixtures. As shown in Table 4, two types of IBPM (IBPM-1 and IBPM-2) were prepared with different compositions determined as described as follows:

- FA and BFS, which are the major components of IBPM concrete, were proportioned to FA:BFS = 4:6 by mass;
- SF, expecting the effect of improving workability of the IBPM concrete, was added to 5% of the binder. This is because the workability of concrete with a low water–binder ratio is possibly improved by adding several percent of SF, which is extremely finer than the main particles, to increase the particle filling rate [17];
- The EX content of 6.0%, in view of the binder content (IBPM content) of around 500 kg/m^3^ of IBPM concrete, was selected for IBPM-1 to be nearly equivalent to the general content of 30 kg/m^3^ for OPC concrete. Moreover, the EX content of IBPM-2 was 11.1%, which was twice that of IBPM-1, to investigate its effect on the compressive strength of IBPM concrete;
- The content of CH (as an alkali stimulant) was around 4.0% in order to achieve good strength development based on the test results of IBPM mortar with W/B of 0.34. In this test, the specimens were subjected to steam curing, shown in Section 2.3, and the results are shown in Figure 3.

### 2.2. Mix Proportions of IBPM Concrete

Table 5 shows the mix proportions of IBPM concrete. The IBPM column in this table represents the unit content of IBPM-1 or -2 detailed in Table 4, which is considered the binder content (B). In conventional cement concrete, the lower the water-to-binder ratio (B/W), the greater the binder content, and the more likely cracks occur in the hardened concrete due to heat of hydration and shrinkage. Therefore, as shown in Table 5, an attempt was made to keep the unit water content (W) as low as possible to minimize the increase in binder content in mixtures with low W/B. As a result, it was confirmed that workability suitable for placement and compaction can be secured even with mixtures with W reduced to 105 kg/m^3^. Furthermore, it was also found that the proper workability for the executions of Hume pipes and box culverts could be obtained with IBPM concrete, which is described in Section 5.1.

### 2.3. Mixing and Curing Procedures

**Figure 4:**
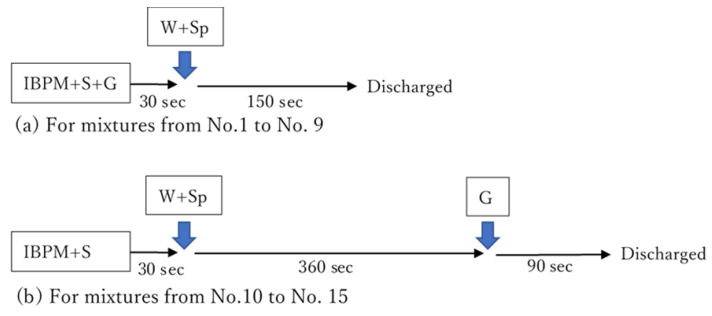
Mixing procedures for IBPM concrete.

Mixing of IBPM concrete was conducted with a biaxial forced mixer with a capacity of 60 L, as shown in Figure 4. For mixtures no. 1 to no. 9, coarse and fine aggregates (G and S) were dry-mixed with IBPM in the mixer for 30 s. Water (W) and superplasticizer (Sp) were then added and mixed for 150 s. For producing IBPM concrete with appropriate workability, this procedure was not enough for mixtures no. 10 to no. 15 with low W/Bs of 19.1 to 24.0%, and with water content being drastically reduced to around 105–120 kg/m^3^. Coarse aggregate was, therefore, added after the mortar attained sufficient workability, and was then further mixed. It took approximately 8 min from the beginning to the end of mixing.

From the experimental results reported in a previous study [18], it was found that high-temperature steam curing, which is generally used in precast concrete factories, is effective at developing the strength of IBPM concrete and that the pre-curing time of steam curing should be slightly longer, i.e., 6 h for IBPM concrete than for conventional cement concrete. Therefore, steam curing under conditions given in Table 6 was applied to IBPM concrete for the specimens for compressive strength and durability tests (Section 3 and Section 4) and the precast concrete mock-up specimens for load-bearing tests (Section 5). 

## 3. Compressive Strength of IBPM Concrete

### 3.1. Development of Compressive Strength

According to previous studies [19], the compressive strength of IBPM concrete (W/B = 0.34), under standard under-water curing at 20 °C, was only about 3 N/mm^2^ at the age of 1 day. As a result, the concrete was still in a fragile state and adhered to the surface of the formwork, causing partial separation, and making it difficult to remove the formwork. On the other hand, in the case of steam curing, the compressive strength was 33 N/mm^2^ at the age of 1 day after the completion of steam curing [19]. Based on this, it was found that proper strength development of IBPM can be obtained by steam curing, and sufficient strength can be secured for demolding at the age of 1 day.

**Figure 5:**
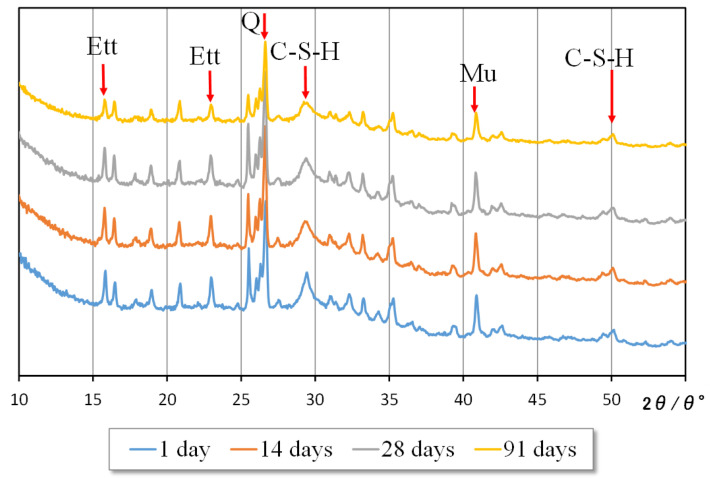
Results of powder X-ray diffraction.

In this study, the hydration products were identified by powder X-ray diffractometry (XRD) using IBPM paste with a W/B of 0.3, where B is the total mass of IBPM. Note that the IBPM paste specimens were steam-cured and then air-cured until the age of 91 days. Figure 5 shows the XRD results for the samples taken immediately after steam curing (1 day of age) and the samples air-cured until the specified ages (14, 28, and 91 days) after steam curing. This figure reveals the peaks of calcium silicate hydrates (C-S-H) and ettringite (Ett), with quartz (Q) and mullite (Mu) also identified. This suggests that the generation of C-S-H and ettringite derived from EX during the hardening process contributed to the strength development of IBPM paste. It was also found that hydration products similar to those at 91 days were formed immediately after steam curing. This result may suggest that steam curing is effective for accelerating the early development of compressive strength of IBPM concrete.

**Figure 6:**
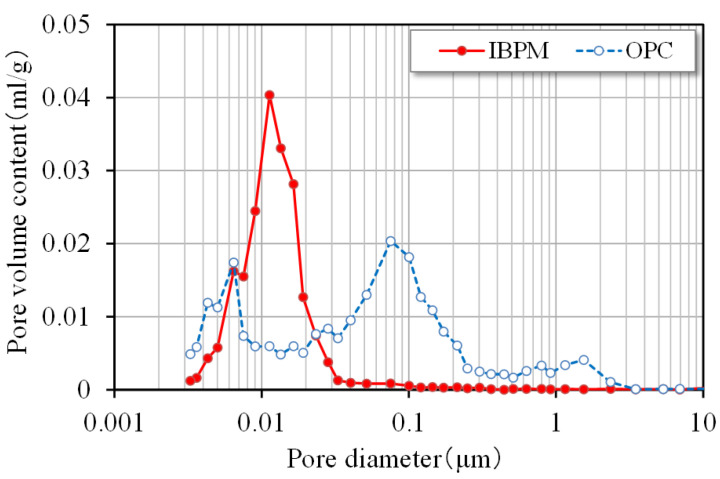
Pore size distributions at 14 days.

**Figure 7:**
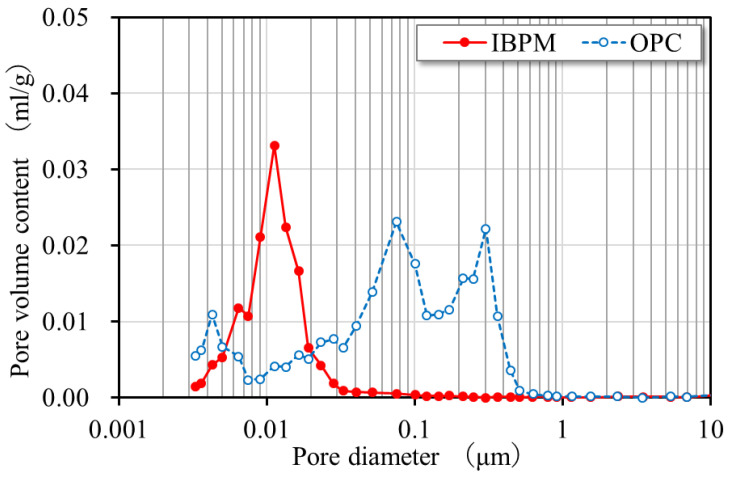
Pore size distributions at 91 days.

Pore size distributions of the IBPM paste and OPC paste were measured by a mercury intrusion porosimeter using the above-mentioned paste specimens. As shown in Figure 6 and Figure 7, a considerable number of pores larger than 0.03 μm existed in the OPC sample. On the other hand, in the IBPM sample, pores larger than 0.03 μm were scarcely recognized but a significantly large number of fine pores at around 0.01 μm existed. The total pore volumes of IBPM and OPC were 0.211 and 0.244 mL/g, respectively, at 14 days, whereas those of IBPM and OPC at 91 days were 0.169 and 0.255 mL/g, respectively. Therefore, the pore structure of the IBPM sample was considered to be denser than that of OPC. Accordingly, it is presumed that a high-temperature history by steam curing activates the pozzolanic reaction of FA and the latent hydraulicity of BFS to form a dense structure in hardened IBPM cement paste. It may be also suggested that the dense microstructure of the IBPM hydration product may contribute to strength development.

### 3.2. Effect of the Water–Binder Ratio on Compressive Strength

A compressive strength test of IBPM concrete was carried out using cylindrical specimens (100 mm in diameter and 200 mm high). Specimens were subjected to steam curing as given in Table 6, demolded at the age of day 1, and then cured in a room at 20 °C, until day 14. This curing procedure is typical for precast concrete products with normal cement, except it involves a slightly longer pre-curing time for steam curing, as described in Section 2.3.

**Figure 8:**
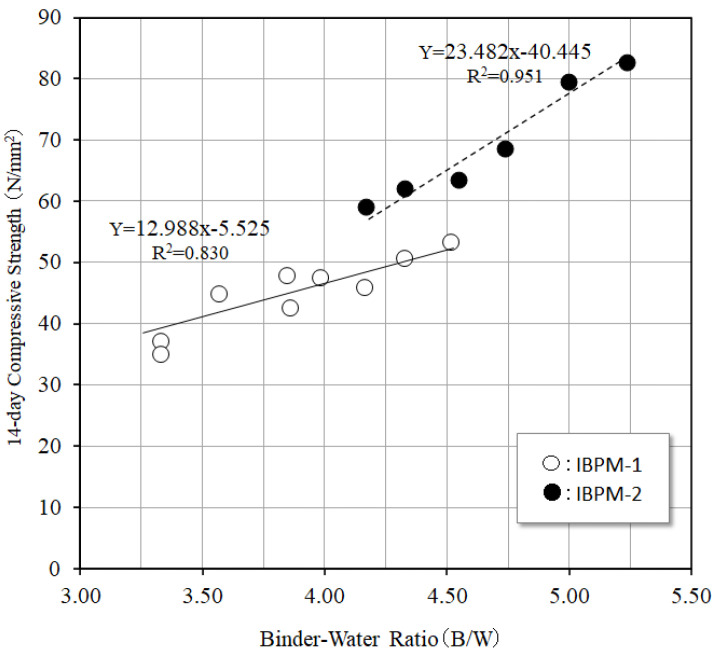
Relationship between the B/W and compressive strength of IBPM concrete.

Figure 8 shows the test results for the relationship between compressive strength at 14 days and the binder–water ratio (B/W) of IBPM concrete proportioned, as given in Table 5. It can be seen from the test results that the compressive strength of IBPM-2 concrete with a higher EX content tends to be higher when compared with IBPM-1 concrete at the same B/W. The effect of EX on compressive strength of alkali-activated material was also reported by other researchers, who demonstrated that EX accelerated the initial reactivity [20]. It was also found feasible to produce IBPM-2 concrete with a compressive strength of up to 80 N/mm^2^ by reducing the water–binder ratio (W/B) to around 0.2, corresponding to a B/W of 5.0. Figure 8 also reveals that the relationship between the B/W and compressive strength of IBPM concrete can be roughly approximated by two different straight lines, which are indicated by the equations in the figure, according to the difference in the EX content (IBPM-1 and IBPM-2). Previous studies have also demonstrated that the water–binder ratio is one of the dominant factors of the compressive strength of alkali-activated materials [3,4]. Therefore, it is considered that, for IBPM concrete with a compressive strength range of 40 to 80 N/mm^2^, the mix proportion design based on compressive strength can be conducted using the fitting lines shown in Figure 8, according to the composition of IBPM (EX content), as is the case for normal cement concrete. Therefore, it can be said that IBPM concrete can possibly be applied to various types of steam-cured precast concrete products with strengths ranging from 40 to 80 N/mm^2^.

## 4. Durability of IBPM Concrete

### 4.1. Chloride Ion-Shielding Property

The chloride ion-shielding property of concrete is important for precast concrete products in a chloride-laden environment, such as box culverts constructed underground along the coast. In this study, cylindrical specimens (50 mm in diameter and 100 mm in length) that were drilled from the mock-up specimens for box culverts were used. The details of the mock-up specimen are shown in Section 5.3. IBPM concrete with a W/B of 21.1%, i.e., mixture no. 13, is shown in Table 5, and was used for the tests. For comparison, specimens were also drilled from box culverts made of OPC concrete (W/B = 38%, s/a = 45%, W = 160 kg/m^3^, C = 420 kg/m^3^, S = 810 kg/m^3^, G = 1009 kg/m^2^, and Sp = 4.20 kg/m^3^). The drilled core specimens were immersed in an aquatic solution with a chloride ion concentration of 10%. Note that the 14-day compressive strengths of IBPM and OPC concretes, which were steam-cured as given in Table 6, and then air-cured, were similar at 59.9 and 55.5 N/mm^2^, respectively.

**Figure 9:**
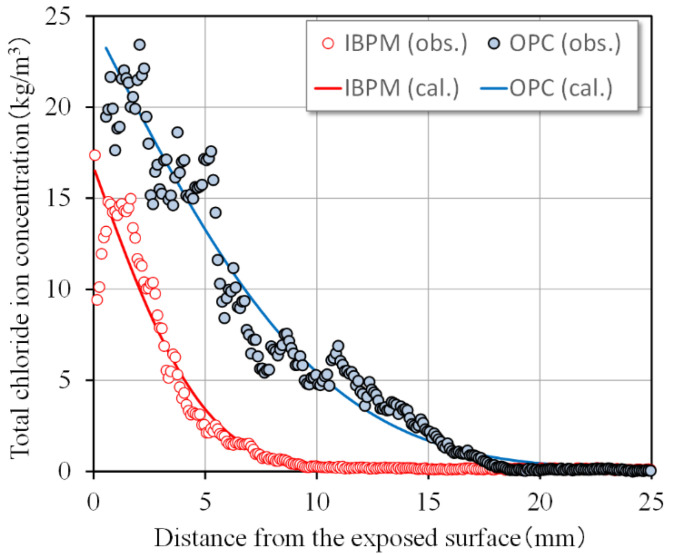
Chloride ion concentration profile in concrete.

Figure 9 shows the profiles of chloride ion concentration after immersion for 3 months evaluated using EPMA in accordance with the JSCE Standard [21]. In this figure, the horizontal axis is the distance from the surface exposed to the solution with chloride ions. The chloride ion concentration of IBPM concrete was as low as around 15 kg/m^3^ at the maximum, with the penetration depth being 9 mm or less, whereas that of OPC concrete was 24 kg/m^3^ at the maximum, and the penetration was around 18 mm. In accordance with the standard method [22,23], the observed distribution of chloride ion was subjected to regression analysis using Equation (1), which is the solution of the diffusion equation based on Fick’s second law, to obtain the apparent diffusion coefficient denoted by constant *D* in the equation.

(1) $$C\left(x\right)=C_{0}\left\{1-erf\left(\frac{x}{2\sqrt{D\cdot t}}\right)\right\}$$

where $C\left(x\right)$ is the total chloride ion concentration at distance *x* (cm) from the exposed surface, $C_{0}$ is the surface chloride ion concentration, t is the immersion period (year), D is the apparent diffusion coefficient, and *erf* is the error function.

The fitting curves by Equation (1) are also shown in Figure 9 and the calculated diffusion coefficients of chloride ions in IBPM and OPC concrete were 0.237 and 1.319 cm^2^/year, respectively. The diffusion coefficient of IBPM concrete was, thus, found to be significantly low, approximately one-fifth of that of OPC. It is suggested that the denser microstructure of IBPM, which is discussed in Section 3.1, may cause better performance against chloride ion penetration than OPC concrete. It can be said from these experimental results that IBPM concrete performs excellent when protecting embedded steel bars in an environment susceptible to salt damage when used for reinforced concrete structures.

### 4.2. Resistance to Sulfuric Acid

Resistance to the sulfuric acid of concrete is important for precast concrete products, such as sewer pipes, which are subject to deterioration by sulfuric acid. This is a phenomenon in which sulfate in sewer pipes changes to hydrogen sulfide and then to sulfuric acid due to the bacterial action, causing the concrete to deteriorate. In order to evaluate the resistance to sulfuric acid of Hume pipes, tubular specimens with IBPM and OPC concrete were fabricated by vibratory or centrifugal compaction and subjected to steam curing under the condition given in Table 6. According to the standard test method for cement concrete sewer pipes [24], the concrete specimens were then immersed in 5% sulfuric acid aqueous solution for 112 days. IBPM concrete proportioned as mixture No. 12, as given in Table 5, was chosen to prepare the specimens for Hume pipes A. Then, the tubular specimens (with 120 mm of internal diameter, 200 mm of external diameter, and 300 mm in length) were fabricated by centrifugal compaction and longitudinally cut into four pieces. One of the pieces was taken for the tests, which involved all the surfaces except the inner surface coated with epoxy and immersed in the sulfuric acid aqueous solution for 112 days. For comparison, the same-sized tubular specimens with OPC concrete (W/B = 0.36, s/a = 41%, W = 158 kg/m^3^, C = 440 kg/m^3^, S = 734 kg/m^3^, G = 1076 kg/m^3^, and Sp = 3.52 kg/m^3^) were also prepared. It should be noted that the 14-day compressive strengths of IBPM and OPC concrete were 71.3 and 53.2 N/mm^2^, respectively.

**Figure 10:**
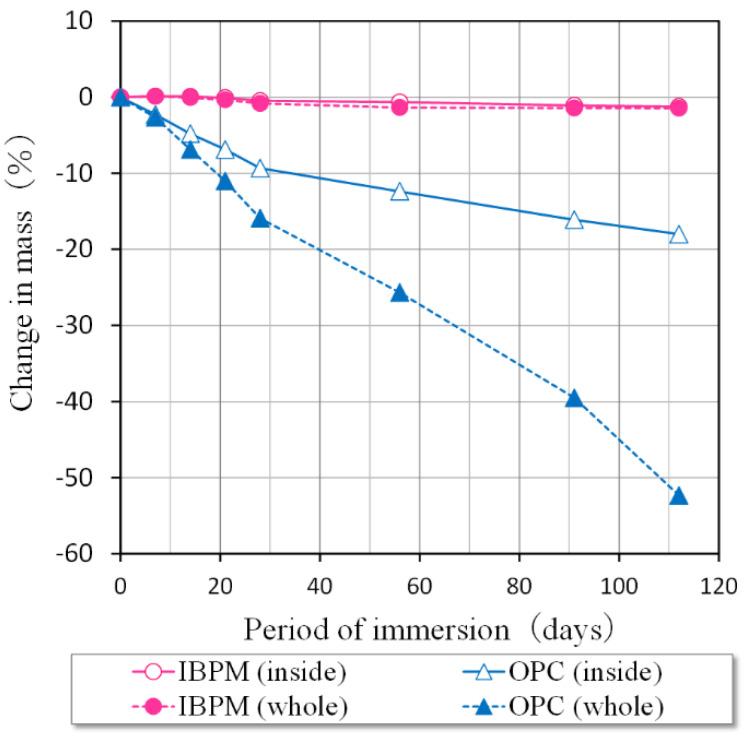
Change in mass of the centrifugally molded specimens by immersion in a sulfuric acid aqueous solution.

**Scheme 1:**
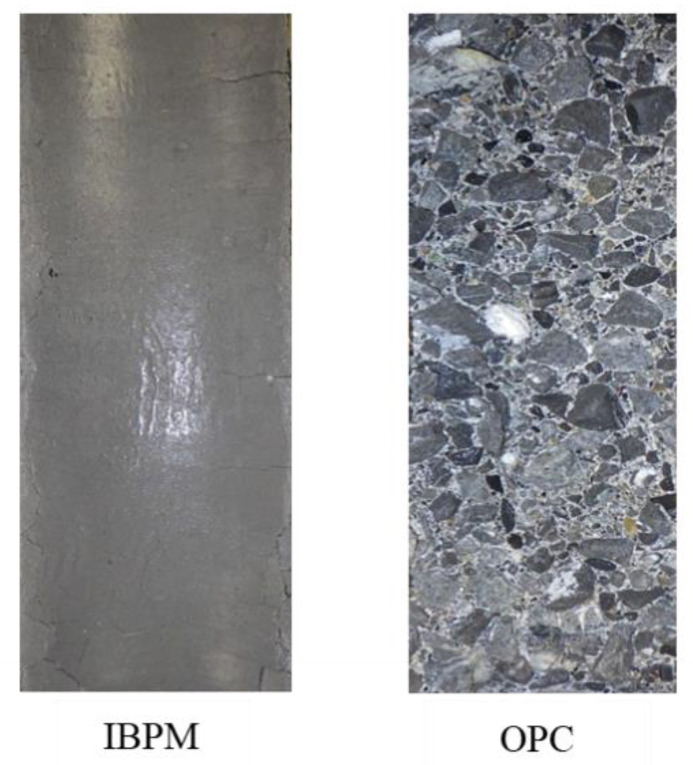
The inner surfaces of the centrifugally molded specimens after immersion in a sulfuric acid aqueous solution.

Figure 10 shows the changes in mass of the specimens due to immersion in the sulfuric acid aqueous solution. The graph legend “inside” in this figure means that only the inner surfaces of the tubular specimens were in contact with the sulfuric acid aqueous solution, and “whole” means that all of the specimen surfaces were. For reference, this figure also shows the test results for the whole tubular specimens without epoxy coating reported in a published paper [25]. The mass losses from the inner surfaces and all the surfaces of IBPM specimens were as low as (about) 1%, although the mass losses of OPC specimens were significantly large. Moreover, as shown in Scheme 1, IBPM concrete remained generally sound even after the immersion tests for 112 days. In contrast, in OPC specimens, the mortar phase eroded, the coarse aggregate was exposed, and deterioration significantly progressed.

**Figure 11:**
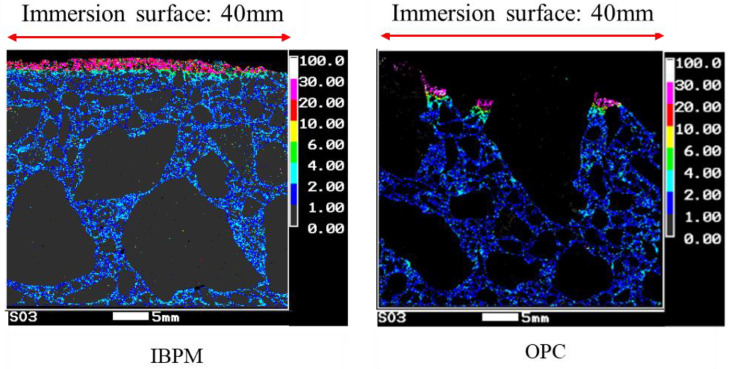
EPMA image of the test pieces near the exposed surface.

After immersion in the sulfuric acid aqueous solution for 112 days, the test pieces measuring 40 by 40 by 20 mm were cut from the inner surfaces of the specimens to measure the depths of the scaled portions. Since some particles of coarse aggregates were exposed on the surface of the severely deteriorated OPC concrete specimens, the depth of the scaled portion of the OPC test piece was measured on the surface of the mortar phase between coarse aggregate particles. The depth of the sulfur penetration was also examined by EPMA using the same test pieces as those for measurements of the scaled portions. As shown in Figure 11 and Table 7, the depth of the scaled portion and sulfur penetration of IBPM concrete were significantly smaller than those of OPC concrete. These experimental results clearly show that IBPM concrete has better performance regarding chloride ion diffusion and sulfuric acid resistance than OPC concrete. As reported in a published paper [19], the other durability performances, such as freeze–thaw resistance and abrasion resistance of IBPM concrete are equivalent to those of OPC concrete.

## 5. Application of IBPM Concrete to Precast Concrete Products

### 5.1. Outline

In the previous section, the compressive strength and durability of IBPM concrete were discussed based on experiments, and the possibility of application to precast concrete products was suggested. Therefore, mock-up specimens of the box culvert and two kinds of Hume pipes were produced at a precast concrete product factory. During the production of the mock-up specimens with IBPM concrete, it was confirmed that placement, compaction, and surface finishing could be properly conducted with the same equipment and procedures as those for mock-up specimens with ordinary Portland cement. In the following sections, the load-bearing capacities of IBPM precast concrete products are demonstrated based on the experiments with full-scale specimens of a box culvert and two kinds of Hume pipes.

### 5.2. Hume Pipes

There are two kinds of construction methods for Hume pipes, the open-cut method and the jacking method. The former is a method in which a trench is excavated and pipes are laid therein, whereas the latter is a trenchless method in which pipes are pushed into the soil from a working well with a jacking system, minimizing traffic restriction. In this study, mock-up specimens of Hume pipe A (for open-cut construction) and Hume pipe B (for jacking construction) were fabricated at a precast concrete plant. Based on the relationship between the B/W and compressive strength of IBPM concrete previously mentioned, the W/B of IBPM concrete for Hume pipes A and B were determined to be 0.26 and 0.22, respectively, to obtain each target of compressive strength.

**Figure 12:**
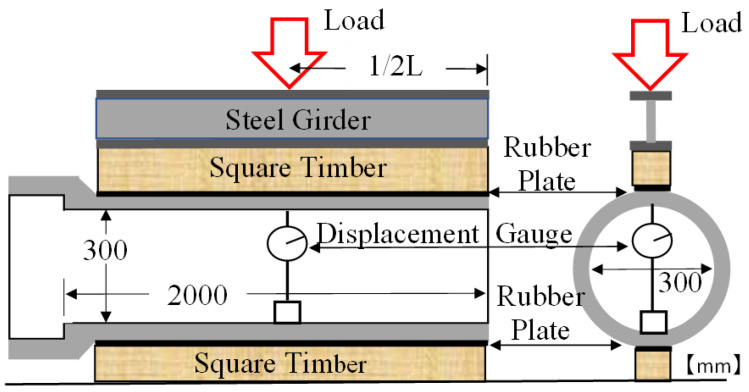
Loading test for mock-up specimen (Hume pipe A).

**Figure 13:**
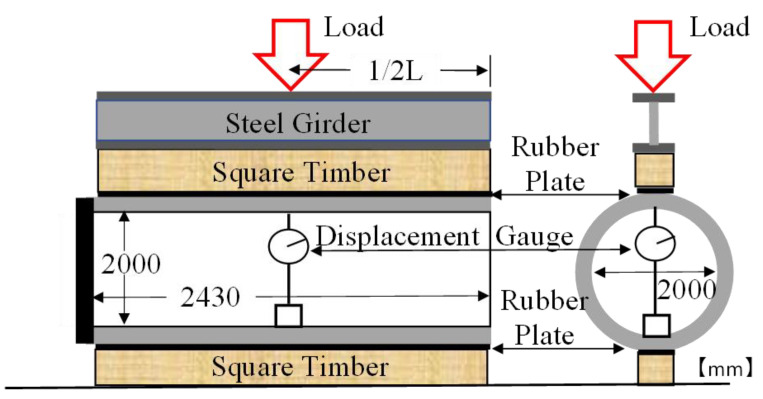
Loading test for mock-up specimen (Hume pipe B).

The mock-up specimen for Hume pipe A has dimensions of 300 mm of internal diameter, 30 mm of thickness, and 2000 mm in length, as shown in Figure 12. Reinforced steel consisted of spirals that were 2.6 mm in diameter at a 44 mm pitch; 8 equally spaced longitudinal straight bars that were 3.2 mm in diameter were arranged in the specimens. The mock-up specimen for Hume pipe B had dimensions of 2000 mm of internal diameter, 175 mm of thickness, and 2340 mm in length, as shown in Figure 13. The reinforcing steel consisted of spirals that were 6.0 mm in diameter at a 60 mm pitch and 24 equally spaced longitudinal straight bars that were 6.0 mm in diameter, which were arranged in the specimens. Table 8 presents the conditions for the compaction of concrete by centrifugal force, in which the cylindrical mold with fresh concrete was rotated at a high speed to consolidate the concrete, while the excessive water was smoothly discharged from the mixture as sludge water and the inner surface was thoroughly consolidated after finishing the surface. After casting, the specimens were subjected to steam curing as presented in Table 6, and stored in air at room temperature until testing.

**Figure 14:**
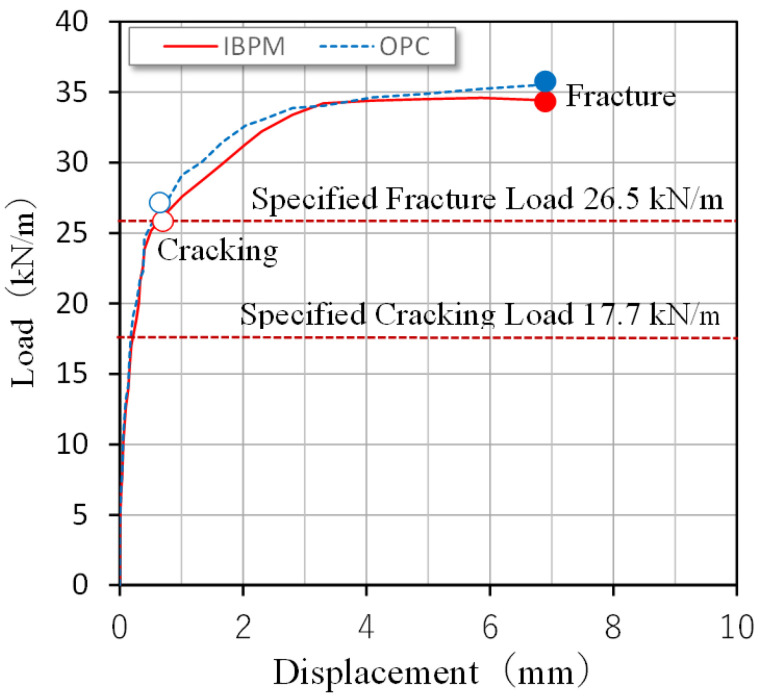
Road versus displacement relationship (Hume pipe A).

**Figure 15:**
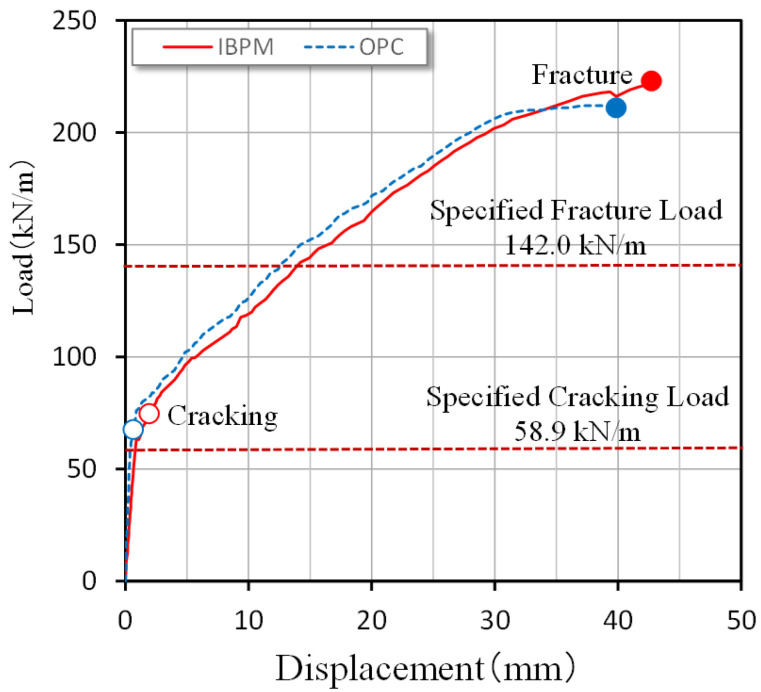
Road versus displacement relationship (Hume pipe B).

The load-bearing performance of the mock-up specimens of Hume pipe A and Hume pipe B were evaluated by static loading tests in accordance with Japanese standards, JIS A 5372 [26], JSWAS A-1 [27], and JSWAS A-2 [28], as shown in Figure 12 and Figure 13, respectively. For comparison, mock-up specimens of Hume pipes A and B were also fabricated using OPC concrete with compressive strengths of 46.9 and 57.0 N/mm^2^, respectively. The judgment criterion for the cracking load was the load at which a crack of 0.05 mm in width was visually recognized. The fracture load was the load at a point at which an extra load no longer increased during the loading tests. Table 9 shows the results of the loading tests for the mock-up specimens made with IBPM and OPC. As a result, both the cracking load and fracture load of Hume pipes A and Hume pipes B made of IBPM concrete comfortably exceeded the required values and were equivalent to those of the specimen with OPC. Moreover, Figure 14 and Figure 15 reveal that the relationships between the load and vertical displacement of the mock-up specimens show nearly the same tendencies. Accordingly, Fume pipes made of IBPM concrete were found to possess load-bearing and deformation performances comparable to those made of OPC concrete.

### 5.3. Box Culverts

**Figure 16:**
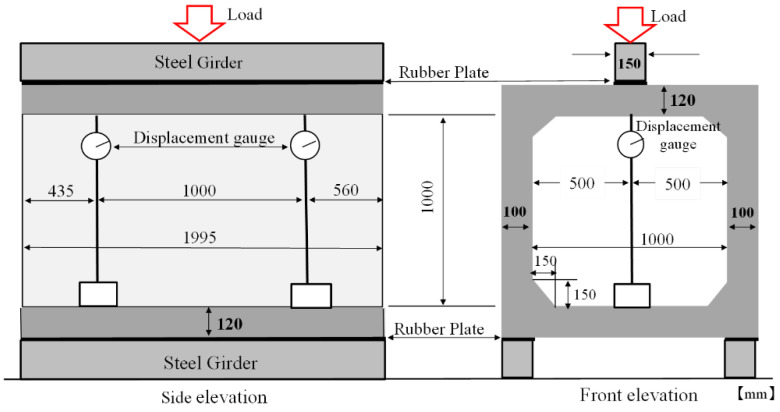
Loading test for mock-up specimen of the box culvert.

Mock-up specimens for box culverts were also prepared at a precast concrete plant. Based on the above-mentioned W/B-strength relationship of IBPM concrete, the W/B of IBPM concrete was determined to be 0.21. Figure 16 shows the geometry of the mock-up specimen of a box culvert with a cross-sectional size of 1000 by 1000 mm and a length of 1995 mm. Deformed bars of 16 and 10 mm in diameter were arranged at 125 mm intervals on the inner and outer sides, respectively, of the culvert walls. The mock-up specimens were subjected to steam curing under conditions given in Table 6 and stored in air at room temperature until the testing.

**Figure 17:**
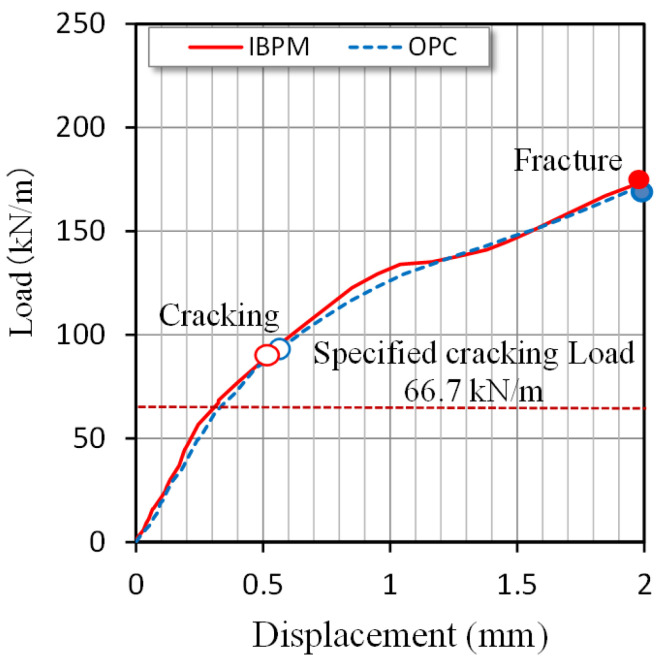
Road versus displacement relationship (box culvert).

The load-bearing performances of the mock-up specimens of box culverts were evaluated by the tests in accordance with JSWAS A-12 [29] as shown in Figure 16. For comparison, mock-up specimens of box culverts were also prepared using OPC concrete with a compressive strength of 55.5 N/mm^2^. It can be seen from Table 10 that the cracking load of the box culverts made of IBPM concrete satisfied the required values and were also equivalent to those of OPC concrete. The test results can be expected because the observed values of the tensile and flexural strengths of each concrete were about the same (see Table 10). As shown in Figure 17, the relationships between the load and the vertical displacement at the loading point of IBPM and OPC box culverts have nearly the same tendencies. This may be because the values of the elastic modulus of IBPM and OPC concrete were almost the same, as shown in Table 10, and as a result, the rigidities of the box culverts with IBPM and OPC concrete should be equivalent. Therefore, it can be seen that box culverts made of IBPM concrete had load-bearing capacities and deformation performances comparable to those made of OPC concrete.

### 5.4. CO_2_ Emission of Precast Concrete with IBPM

In order to estimate the CO_2_ emissions from the production of IBPM concrete in comparison to that of OPC concrete, trial calculations were carried out for the box culvert and Hume pipe. In the calculations, the CO_2_ emission intensities of each material shown in Table 11 were used, and those of OPC, fine aggregate, and coarse aggregate were 767.3, 2.9, and 3.7 kg-CO_2_/t, respectively [30]. It can be seen from the table that the calculated CO_2_ emission of IBPM-2 was 136.9 kg-CO_2_/t, which was much less than that of OPC. Then, the CO_2_ emissions of the box culvert and Hume pipe B were calculated based on their mix proportions of concrete, which are shown in Table 5 for IBPM concrete and in Section 4.1 and Section 4.2 for OPC concretes, respectively. The calculated CO_2_ emission per 1 m^3^ of IBPM concrete was 81.2 kg for the box culvert and 73.2 kg for Hume pipe B. These values were only 25% and 21% of those of the corresponding products with OPC concrete, respectively. Since steam curing is generally applied to precast concrete products regardless of the binder type, the CO_2_ emission due to energy consumption of the steam curing was also considered. When the CO_2_ emission intensity of heavy oil was assumed to be 2.91 kg-CO_2_/L [31], the ratios were 33% and 30%, respectively. It was suggested from these trail calculations that IBPM concrete could be effectively used to reduce CO_2_ emissions of precast concrete products.

## 6. Conclusions

In this study, cementless binder (mainly comprising fly ash and ground granulated blast furnace slag, with the addition of calcium hydroxide as a stimulant (IBPM)) was proposed as material for steam-cured precast concrete products. Within the range of the experimental conditions, the findings are summarized as follows.

1. It is proved that IBPM concrete with compressive strength from 40 to 80 N/mm^2^ can be produced by high-temperature steam curing. The compressive strength of IBPM concrete is related to its binder–water ratio, with the relationships expressed by different straight lines depending on the expansive additive content.
2. Increases in the binder content in IBPM concrete mixtures with decreases in the water–binder ratio can be suppressed by reducing the unit water content down to around 105–120 kg/m^3^.
3. The chloride ion diffusion coefficient of IBPM concrete is approximately one-fifth of that of OPC concrete.
4. The inner surface of the Hume pipe with IBPM concrete made by the centrifugal compaction method rarely suffers deterioration by sulfuric acid.
5. From the test results of static loading on the mock-up specimens, it is suggested that precast concrete box culverts and Hume pipes with IBPM concrete have almost the same load-bearing capacities and deformation performances as those of OPC concrete.

## Figures and Tables

**Table 1:** Materials for IBPM.

Material	Symbol	Density (g/cm^3^)	Specific Surface Area (cm^2^/g)	Activity Index at 28 Days (%)
Fly ash	FA	2.20	3480	81
Ground granulated blast furnace slag	BFS	2.91	4230	92
Silica fume	SF	2.30	191,000	110
Expansive additive	EX	3.15	4560	-
Calcium hydroxide	CH	2.32	150,000	-

**Table 2:** Chemical Compositions of Materials.

Material	Ig. Loss (%)	Chemical Composition (%)									
		SiO_2_	Al_2_O_3_	Fe_2_O_3_	CaO	MgO	SO_3_	Na_2_O_3_	K_2_O	TiO_2_	P_2_O_5_
FA	2.63	52.78	29.05	4.12	3.79	1.90	0.26	0.50	1.09	1.59	0.41
BFS	0.08	34.03	14.69	0.41	42.90	6.01	-	0.22	0.32	0.55	0.01
SF	3.71	92.97	-	-	-	0.37	0.65	-	-	-	-
EX	4.40	4.43	1.36	0.67	60.36	0.64	27.28	0.12	0.11	0.07	0.11
CH	24.76	0.19	0.11	0.12	73.82	0.77	0.07	ND	0.01	0.02	0.03

**Table 3:** Aggregate properties for IBPM concrete.

Classification	Kind	Density (g/cm^3^)	Absorption (%)	Solid Content (%)	Fineness Modulus	Rock Type
Fine aggregate	Crushed sand	2.62	1.76	65.5	2.64	Tight sandstone
Coarse aggregate	Crushed stone	2.67	1.51	58.2	6.69	

**Table 4:** Blend ratio of IBPMs.

Type	Blend Ratio (%)					
	FA	BFS	SF	EX	CH	Total
IBPM-1	33.0	51.0	6.0	6.0	4.0	100.0
IBPM-2	31.3	48.2	5.6	11.1	3.8	100.0

**Table 5:** Mixed proportions of IBPM concrete.

No.	IBPM Type	W/B	s/a (%)	Unit Content (kg/m^3^)					Air (%)	Slump Flow/ (Slump) (cm)	Remark
				W	IBPM	S	G	Sp			
1	1	0.300	45	150	500	750	934	6.00	1.6	53.5	-
2	1	0.300	41	120	400	757	1111	5.20	1.6	(16.5)	-
3	1	0.280	45	112	400	841	1066	5.60	1.7	48.0	-
4	1	0.260	45	130	500	773	982	5.00	1.4	54.5	-
5	1	0.259	41	114	440	747	1095	6.60	1.4	(8.0)	Hume Pipe A
6	1	0.251	41	138	550	678	994	6.60	1.6	54.0	-
7	1	0.240	41	132	550	681	1001	6.60	1.7	53.5	-
8	1	0.231	41	127	550	686	1009	6.60	1.8	53.0	-
9	1	0.221	41	124	560	686	1007	7.28	1.6	53.5	-
10	2	0.240	41	120	500	715	1049	8.00	1.5	62.0	-
11	2	0.231	41	120	520	707	1036	8.58	1.4	63.5	-
12	2	0.220	41	108	490	734	1076	7.84	1.4	55.5	Hume Pipe B
13	2	0.211	45	116	550	770	959	10.18	1.5	58.0	Box Culvert
14	2	0.200	41	110	550	707	1039	9.90	1.7	55.5	-
15	2	0.191	41	105	550	713	1046	11.83	1.9	54.0	-

**Table 6:** Steam curing conditions.

Item	Set Condition
Pre-curing time	6 h
Heating rate	20 °C/h
Maximum temperature	65 °C
Maximum temperature-keeping time	4 h
Cooling conditions	Natural cooling

**Table 7:** Sulfuric acid resistance of the internal portion of centrifugally molded specimens.

Binder	Thickness of Specimen (mm)		Depth of Scaled Portion (mm)	Sulfur Penetration (mm)
	Before Immersion	After Immersion		
IBPM	40.5	39.0	1.5	7
OPC	40.4	33.3	7.1	15

**Table 8:** Centrifugal molding conditions.

Item	First Stage	Second Stage	Third Stage
Acceleration G: gravitational acceleration	5 G	15 G	35 G
Centrifugal compaction time	1 min	1 min	7 min

**Table 9:** Load-bearing capacities of Hume pipe specimens and compressive concrete strengths.

Products	Item	Cracking Load (kN/m)	Fracture Load (kN/m)	Compressive Strength (N/mm^2^)
Hume pipe A	Specified value	17.7	26.5	-
	Observed (IBPM)	26.1	34.6	45.6
	Observed (OPC)	27.9	35.6	46.5
Hume pipe B	Specified value	58.9	142.0	-
	Observed (IBPM)	65.4	222.2	58.2
	Observed (OPC)	59.3	211.0	56.7

**Table 10:** Load-bearing capacities of box culvert specimens and mechanical properties of concrete.

Item	Cracking Load (kN/m)	Fracture Load (kN/m)	Compressive Strength (N/mm^2^)	Tensile Strength (N/mm^2^)	Flexural Strength (N/mm^2^)	Modulus of Elasticity (kN/mm^2^)
Specified value	66.7	-	50.0	-	-	-
Observed (IBPM)	96.2	399.4	59.9	4.1	5.4	35.3
Observed (OPC)	96.9	392.0	55.5	3.8	5.4	33.4

**Table 11:** Estimated CO_2_ emissions of IBPM-2.

Materials	CO_2_ Emission Intensity (kg-CO_2_/t)	Blend Ratio (%)	CO_2_ Emission (kg-CO_2_/t)
FA	19.6	31.3	6.1
BFS	26.5	48.2	12.8
SF	19.6	5.6	1.1
EX	764.3	11.1	84.8
CH	844.6	3.8	32.1
Total	2.9	100.0	136.9

## Data Availability

Not applicable.
